# Society Meetings

**Published:** 1904-06

**Authors:** 


### SOCIETY MEETINGS.

The Buffalo Academy of Medicine held meetings during the months of April and May as follows:

*Section on Obstetrics and Gynecology.*—Tuesday evening, April 26. Program: The relation between neurasthenia and diseases of the female generative organs, Herman B. Singer.

*Section on Surgery.*—Monday evening, May 2. Program: The *x*-ray in diagnosis and treatment; presentation of cases; suggestions for treatment of *x*-ray burns, Sidney A. Dunham; discussion by Drs. Bayliss, Preiss and O'Gorman.

*Section on Medicine.*—Tuesday evening, May 10. Program: Spinal cord tumors, William C. Krauss; discussion by Floyd S. Crego. Recurrent vomiting in children, Irving M. Snow; discussion by Eli H. Long.

*Section on Pathology.*—Tuesday evening, May 17. Program: Observation on intestinal parasites in troops returned from the Philippine Islands, with demonstrations, H. M. Hallock, Captain U. S. A. Short communications and demonstrations by N. G. Russell, Irving M. Snow, Harvey R. Gaylord, and G. H. A. Clowes.

---

The following societies will meet at Atlantic City, during early June, 1904, namely: American Medical Association, June 7-10; American Academy of Medicine, June 4-6; American Gastro-enterological Association, June 6; Association of American Medical Colleges, June 7; American Medical Editors' Association, June 6; American Orthopedic Association, June 7-9; American Urological Association, June 10-11; National Association of United States Pension Examining Surgeons, June 6-7; National Confederation of State Medical Examining and Licensing Boards, June 6; Medical Society of the State of New Jersey, June 4-7.

---

The American Association of Obstetricians and Gynecologists will hold its seventeenth annual meeting at Saint Louis, Tuesday, Wednesday, Thursday and Friday, September 13, 14, 15 and 16, 1904, under the presidency of Dr. Walter Blackburn Dorsett. The sessions will be held daily from 9.30 a. m. to 2 p. m., with a ten-minute recess at noon. There will be no other sessions during the day or evening. The headquarters will be at the Hotel Monticello. The medical profession is cordially invited.

---

THE Medical Society of the County of Erie will hold its eighty-third semi-annual meeting at the Buffalo Library Building, Tuesday, June 14, 1904, at 10 o'clock a. m., under the presidency of Dr. William C. Krauss, of Buffalo. The program is in preparation and will be issued early in June.

---

THE American Microscopical Society will hold its twenty-fifth annual meeting in Buffalo, August 23, 24 and 25, 1904. The first meeting of this society was held in Buffalo in 1879, which again entertained the society in 1889, and it is therefore fitting that the silver celebration should be held here this year. The coöperation of physicians and scientists is cordially invited. Further particulars of the meeting will be published in the August number of the JOURNAL.

